# Mobile Health for Pediatric Weight Management: Systematic Scoping Review

**DOI:** 10.2196/16214

**Published:** 2020-06-03

**Authors:** Louise Tully, Amanda Burls, Jan Sorensen, Riyad El-Moslemany, Grace O'Malley

**Affiliations:** 1 School of Physiotherapy Division of Population Health Sciences Royal College of Surgeons in Ireland Dublin Ireland; 2 School of Health Sciences Division of Health Services Research and Management City University of London London United Kingdom; 3 Healthcare Outcomes Research Centre Royal College of Surgeons in Ireland Dublin Ireland; 4 Department of Psychology Royal College of Surgeons in Ireland Dublin Ireland; 5 W82GO Child and Adolescent Weight Management Service Children’s Health Ireland at Temple Street Dublin Ireland

**Keywords:** childhood obesity, behavior change, weight management, mHealth, eHealth, connected health, lifestyle medicine, digital health

## Abstract

**Background:**

The prevalence and consequences of obesity among children and adolescents remain a leading global public health concern, and evidence-based, multidisciplinary lifestyle interventions are the cornerstone of treatment. Mobile electronic devices are widely used across socioeconomic categories and may provide a means of extending the reach and efficiency of health care interventions.

**Objective:**

We aimed to synthesize the evidence regarding mobile health (mHealth) for the treatment of childhood overweight and obesity to map the breadth and nature of the literature in this field and describe the characteristics of published studies.

**Methods:**

We conducted a systematic scoping review in line with the Preferred Reporting Items for Systematic Reviews and Meta-Analyses extension for scoping reviews, by searching nine academic databases in addition to gray literature for studies describing acceptability, usability, feasibility, effectiveness, adherence, or cost-effectiveness of interventions assessing mHealth for childhood obesity treatment. We also hand searched the reference lists of relevant articles. Studies aimed at the prevention of overweight or obesity were excluded, as were studies in which mHealth was not the primary mode of treatment delivery for at least one study arm or was not independently assessed. A random portion of all abstracts and full texts was double screened by a second reviewer to ensure consistency. Data were charted according to study characteristics, including design, participants, intervention content, behavior change theory (BCT) underpinning the study, mode of delivery, and outcomes measured.

**Results:**

We identified 42 eligible studies assessing acceptability (n=7), usability (n=2), feasibility or pilot studies (n=15), treatment effect (n=17), and fidelity (n=1). Change in BMI z-scores or percentiles was most commonly measured, among a variety of dietary, physical activity, psychological, and usability or acceptability measures. SMS, mobile apps, and wearable devices made up the majority of mobile interventions, and 69% (29/42) of the studies specified a BCT used.

**Conclusions:**

Pediatric weight management using mHealth is an emerging field, with most work to date aimed at developing and piloting such interventions. Few large trials are published, and these are heterogeneous in nature and rarely reported according to the Consolidated Standards of Reporting Trials for eHealth guidelines. There is an evidence gap in the cost-effectiveness analyses of such studies.

## Introduction

### Background

Childhood obesity remains a leading global public health concern, particularly its prevalence among children because of the short-term comorbidities and long-term impacts on psychological well-being, physical development, risk of noncommunicable disease, progression of comorbidities, and the subsequent economic implications [1]. Although the level of fat accumulation in the body is difficult to measure, widely accepted proxy methods for classifying the level of adiposity, such as age- and gender-adjusted BMI centile curves, are available from the International Obesity Taskforce [2,3], as well as the World Health Organization (WHO) [4] and the Centers for Disease Control and Prevention [5]. During childhood and adolescence, the cornerstone of treatment for overweight and obesity is lifestyle interventions, and the evidence shows behavior change techniques, such as goal setting, incentives, family support, and self-monitoring, alongside dietetic support and increased physical activity to be effective [6-8]. For those with more severe obesity, pharmacotherapy and bariatric surgery may need to be considered [9]. For the remainder of this paper, we discuss lifestyle interventions only when referring to treatment.

The complexity of obesity continues to unfold as researchers and practitioners strive to develop both prevention and treatment options that are effective and sustainable [10]. In doing so, many researchers and practitioners have sought to utilize information and communication technology (ICT) and, in particular, the ubiquity of mobile technology in both developed and developing countries, to deliver treatment with wide reach and efficiency [11]. The WHO Global Observatory for Electronic Health defines mobile health (mHealth) as “medical and public health practice supported by mobile devices, such as mobile phones, patient monitoring devices, personal digital assistants (PDAs), and other wireless devices” [12].

### Related Work

Robust evidence syntheses have shown mHealth interventions to be effective tools in enhancing care for the management of certain chronic diseases, including asthma and diabetes [13,14], and it is important to assess their potential in other populations with chronic diseases. Ambulatory care via mobile devices for pediatric obesity could provide treatment to those less likely to access services because of practicality and geography, or indeed to those who live chaotic lives wherein the capacity to attend clinic appointments is compromised. Social distancing measures currently in place in response to a global pandemic have also introduced an urgent need for alternative options for outpatient consultations. Utilizing ICT also allows for a means of accessing therapeutic care that may help to overcome issues associated with stigma and obesity. There is also the potential for saving considerable staff and patient time, improving health-monitoring data collection quality and consistency, and allowing for increased self-efficacy on the part of the patient in the management of conditions [14]. In addition to the interest in digital health from the health care sector itself, mHealth in the context of childhood obesity has recently become the focus of commercial interest also [15], which further reinforces the need for a review of the published evidence.

Previous use of telehealth for the treatment of childhood obesity has shown to be promising, particularly for reaching rural and less-accessible patients [16], and carefully designed mHealth interventions have the potential for improving this reach, given the increasing popularity of mobile electronic devices. The widespread use of mobile electronic devices, in particular, smart devices (such as mobile phones and tablet PCs), has accelerated in the last decade leading to two-thirds of the world’s population being connected to mobile devices [17]. Further, practitioners involved in pediatric weight management have demonstrated openness to the use of mHealth to support treatment [18].

With this rapid social transition to the use of handheld and mobile technology within all aspects of daily life, there has been a sharp increase in research that incorporates mHealth [19]. Despite this increase, challenges remain with respect to augmenting, complementing, or even substituting face-to-face treatment of overweight and obesity with technology. De Jongh et al [13] highlighted the need for further assessment of long‐term effects, acceptability, costs, and risks of mHealth interventions. The promotion of mobile devices for health care in this population (eg, children aged <12 years) could be viewed as contrary to the WHO [20] and local guidance to minimize screen time for children, and this is a potential source of confusion and perhaps adverse effects.

There is also the consideration of whether transferring face-to-face clinical services to platforms, which rely on considerably expensive devices could negatively impact on existing health inequalities. Ownership of mobile devices is widespread across the various socioeconomic categories; however, it is still possible that this could further isolate the most vulnerable groups living in poverty, a well-documented driver for obesity. Researchers must also be mindful of digital literacy issues and their impact on inequalities. Previous research has suggested that children with the lowest socioeconomic status are likely to benefit the least from obesity prevention interventions [21], and this should also be considered carefully in relation to treatment efforts.

Many potential advantages of using mHealth also need to be balanced with data protection and privacy considerations and protocols, which vary globally [22]. There may be an unintended risk of compromising children’s privacy and safety on the Web by enrolling them in mHealth interventions, particularly if ownership or access to data is not specifically detailed. Potential adverse events concerning physical safety due to distractions from the environment because of mobile phones is also a consideration for those interested in pediatric mHealth interventions.

In addition, those seeking to leverage mobile technology for the improvement of health care should do so while also assessing its cost-effectiveness. The financial resources required to establish, maintain, and future-proof mHealth interventions may be easy to overlook compared with the more obvious and visible resources required to run a physical in-person obesity clinic. The development and maintenance of modern ICT is associated with significant economic, environmental, and ethical costs. The global demand for ICT (data centers, networks, and connected devices) corresponds to substantial global carbon emissions, and its true cost is difficult to measure [23]. Moreover, the sourcing of materials necessary for manufacturing mobile devices are the subject of ethical concern [24]. These fundamental environmental and social costs are in addition to the cost of translating lifestyle interventions to Web-based or mobile platforms, costs related to design, development, and delivery of software, and cost of testing efficacy in robust trials and rolling out interventions (delivery, evaluation, and maintenance). The true impact of seemingly cost-effective alternatives to conventional health care may be substantial.

### Objectives

To date, despite a number of reviews aimed at assessing mHealth for health-promoting behaviors related to the prevention of childhood obesity [11,25], there has not been a review that focused on mobile technology for clinical pediatric weight management. Therefore, we sought to assess what has been researched by mapping the published work and gray literature describing studies of mobile technology for pediatric weight management. We specifically sought to map the methods used and characteristics of studies to present a broad overview of the parameters of work in this field to date and inform future studies that may aim to synthesize findings related to particular outcomes of interest.

This study aimed to assess the breadth and nature of the available literature describing evaluations of interventions using mHealth for the treatment of childhood overweight or obesity.

## Methods

### Design

We used a scoping review methodology [26-29] to achieve the research aim, with the Preferred Reporting Items for Systematic Reviews and Meta-Analyses extension for scoping reviews (PRISMA-ScR) as guidance throughout the reporting process [28]. Our objective was to provide a descriptive map of the characteristics of studies to date in the broad context of mHealth for pediatric weight management, in line with Arksey and O’Malley’s [26] framework for scoping reviews. We sought to include evaluations of mHealth interventions, in addition to studies assessing feasibility, acceptability, adherence, or cost. A protocol for this study was registered on Open Science Framework [30].

### Study Selection

We constructed the search strategy (see Multimedia Appendix 1 for full strategy) iteratively with guidance from a research librarian, adapting this where necessary for the various search engines. There were three components of the search strategy, which incorporated (1) search terms for all related terms to the technology and potential devices used, (2) all search terms which might characterize children (ie, anyone aged <18 years), and (3) all terms identified related to obesity and weight management (Table 1).

We undertook systematic searches of academic databases, including PubMed, Cumulative Index to Nursing and Allied Health Literature, PsycINFO, Health Business Elite, Medical Literature Analysis and Retrieval System Online, Excerpta Medica dataBASE, Cochrane, Emerald, Scopus, and Web of Science, for articles published between January 2000 and December 2018 to ensure the relevant technology was included. In addition, we searched for gray literature using Connecting Repositories [31], OpenGray, Rian and Bielefeld Academic Search Engine, and also by hand searching the reference lists of relevant articles. Articles were limited to those published in English. We included gray literature such as reports, theses, and conference proceedings for completeness (if the same results were not included as a full article) [32].

**Table 1:** Search strategy.

Component number	Search string
1	microcomputer OR telemedicine OR personal digital assistant OR digital health OR wireless OR smartphone OR ‘cell phone’ OR ‘mobile phone’ OR handheld OR mhealth OR app OR tablet computer OR tablet PC OR iPad OR messaging OR messages OR eHealth OR ‘electronic health’ OR telehealth OR connected health OR internet OR (mobile AND app) OR (mobile AND electronic AND device) OR (mobile AND health) OR (mobile AND application)
2	child* OR adolescen* OR teen* OR youth OR minors OR pediatric OR paediatric OR young
3	obesity OR obese OR overweight OR weight loss OR BMI OR “body mass index” OR “body weight” OR “weight management”

The research team carried out two rounds of screening; the first author screened all titles, abstracts and full texts, and a 20.00% (944/4719) portion of titles and abstracts and 25.1% (80/318) full texts were double screened by other reviewers to ensure consistency. Where there were discrepancies between decisions for each stage, both reviewers discussed their decisions and a third researcher was available if needed for consensus.

### Inclusion and Exclusion Criteria

Studies were eligible for inclusion if they assessed lifestyle interventions using mHealth for weight management (ie, lifestyle treatment aimed at reducing adiposity or related clinical measures or maintaining weight following treatment) in children and adolescents ≤18 years with overweight or obesity (as defined by local criteria). We included studies if the mobile component was the primary mode of intervention delivery for at least one study phase, or if the mobile component was independently assessed. Qualitative, quantitative, or mixed and multiple method studies assessing acceptability, usability, feasibility, effectiveness, cost-effectiveness, fidelity, or adherence were eligible to achieve our aim of mapping the breadth and nature of the literature in this field.

We excluded studies aimed at preventing overweight in children of normal weight. We excluded studies in which participants were inpatients, or the intervention was aimed at managing underweight participants. Interventions in which the primary purpose of the mobile technology component was collecting outcome data (eg, a smartwatch to collect data on physical activity) were only included if the intervention also involved tailored feedback or counselling based on the data collected from the device. Studies in which the digital component comprised Web 2.0 platforms that are commonly accessed via apps, but can also be accessed using computers, were only included if the authors specified that only a mobile device would be used by the participants. If it was unclear what type of device would be used, then studies were excluded. The full inclusion and exclusion table used can be found in Multimedia Appendix 2.

### Data-Charting Process

Assessment of eligibility was aided by a decision tool developed for this study (Multimedia Appendix 3), and studies were then categorized according to the characteristics agreed upon a priori [30], using a data extraction form developed for this review. The characteristics were as follows: aim, design, participants, nature of mHealth, the outcomes assessed and measures used, and details of the behavior change theory (BCT, if any) underpinning the mHealth intervention. Data were extracted by the first author using the predefined form.

## Results

### Study Selection

**Figure 1:**
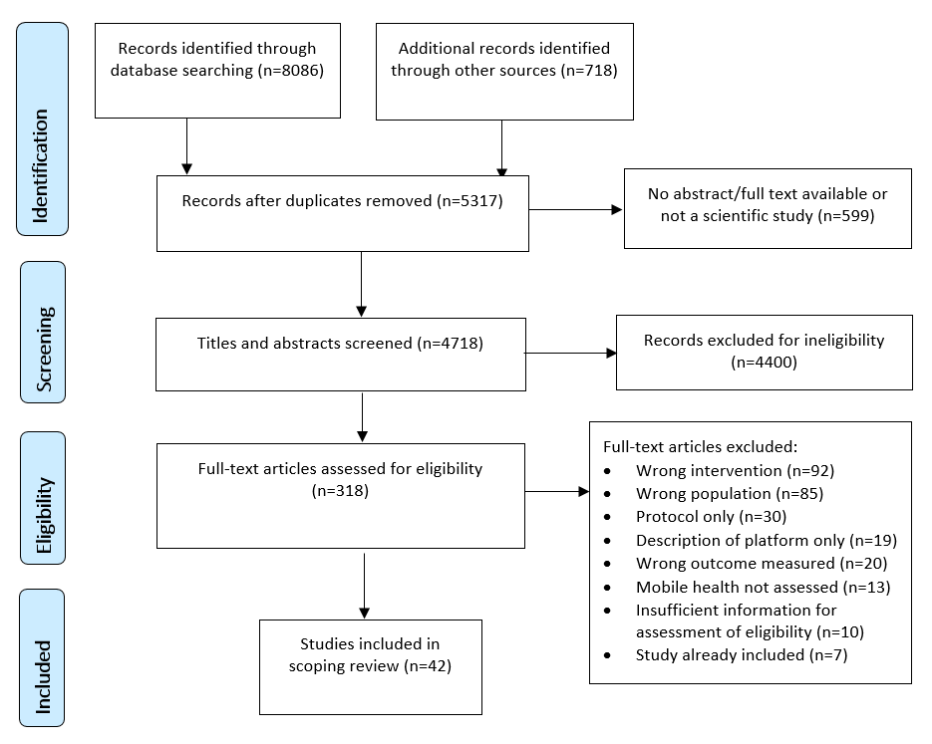
Preferred Reporting Items for Systematic Reviews and Meta-Analyses (PRISMA) flowchart.

We identified 8804 titles through database and gray literature searching (Figure 1), and upon removal of duplicates and other ineligible data sources, we screened 4718 titles and abstracts for eligibility with 318 full texts screened thereafter. The initial agreement between reviewers during the title and abstract screening phase was 86.1% (813/944), and 87.5% (70/80) for the full-text screening. All conflicts were resolved through discussion between the reviewers involved in each stage. In both phases, initial disagreement had been because of over-inclusiveness on the part of the second reviewer, which on a more detailed discussion in the context of the eligibility criteria resulted in complete agreement.

### Nature of the Literature

We identified 42 studies (based on 25 interventions), which met our eligibility criteria (Multimedia Appendix 4) [33-77]. Of our included studies, 32 were published journal articles and 9 were conference proceedings; 1 doctoral thesis was also included. Of the studies included, 7 were aimed at assessing acceptability among participants [33-38,78], and 2 were usability studies [39,40]. In all, 15 were either feasibility [41-45] or pilot [46-55] studies, and 17 reported outcomes of trials or field studies [35,56-72]. We also identified 1 process evaluation [73]. We did not identify any economic evaluations of mHealth for childhood obesity treatment.

Notably, we identified 30 additional research protocols or registered trials (not included in our review) for studies assessing mHealth for childhood obesity treatment, which were ongoing or not yet published. The majority of these (63%, 19/30) are based on mobile apps as the primary mode of delivery, with 17% (5/30) incorporating wearable technology, and 13% (4/30) using SMS as the only mHealth component.

### Participants

The sample size of included studies ranged from 3 to 262 participants. The majority of studies included a small sample size; 45% (19/42) studies included <25 participants, while 67% (28/42) studies included <50 participants). Overall, 29% (12/42) studies included more than 100 participants, and these were all randomized controlled trials (RCTs) (Multimedia Appendix 4), although 3 were pilot or feasibility trials [43,49,55].

All of the interventions aimed at treating childhood obesity and, therefore, included children or adolescents with overweight or obesity. The precise criteria used for the participant inclusion were not always specified, but where they were specified, these were predominantly based on BMI centiles for age and gender, and varied from ≥85th centile to ≥98th centile (see Multimedia Appendix 4). Tripicchio et al [66] included the widest age range and the youngest sample of children, with participants aged 2 to 18 years in a family-based intervention, while Kim et al [38] included the oldest participants, with an age range 13 to 29 years. There were also 3 studies where the intervention focused on the parents [37,59,69]. One additional study aimed at young people with intellectual or developmental disabilities (IDD) [46] targeted parents for a qualitative acceptability study to assess their preferences [78]; however, the main intervention was tested with children and young people [46]. Aside from the studies by Ptomey et al [46,78] for children with IDD, only 1 study had additional inclusion criteria to BMI classification; Patrick et al [60] also specified that participants should have two risk factors for type 2 diabetes mellitus in addition to BMI ≥85th centile. The participant characteristics for each study are also presented in Multimedia Appendix 4.

### Intervention Content

Of all the studies identified, 69% (29/42) specified a BCT, or a component of BCT, on which the intervention being assessed was based (Multimedia Appendix 4). In terms of content, almost all of the interventions were multicomponent and focused on various aspects of lifestyle treatment for obesity (diet and physical activity predominantly), incorporating food or physical activity diaries, games, encouragement or feedback related to adherence to physical activity and nutrition goals, or general motivation. Kulendran et al [49] assessed SMS for weight maintenance by comparing commitment-based techniques with information only.

In the study by Saez et al [43], the SMS intervention was solely aimed at motivating participants to attend face-to-face sessions. One study focused only on diet [47], and 2 on only physical activity [54,67]. Similar to the study by Saez et al [43], the SMS component described by Herget et al [67] was primarily aimed at encouraging attendance at physical activity sessions. Overall, 5 of the included studies assessed interventions based only on the self-monitoring aspect of lifestyle treatment, with the technology aimed at recording diet and physical activity [35,38,45,50,61].

### Modes of Delivery for Mobile Health

**Figure 2:**
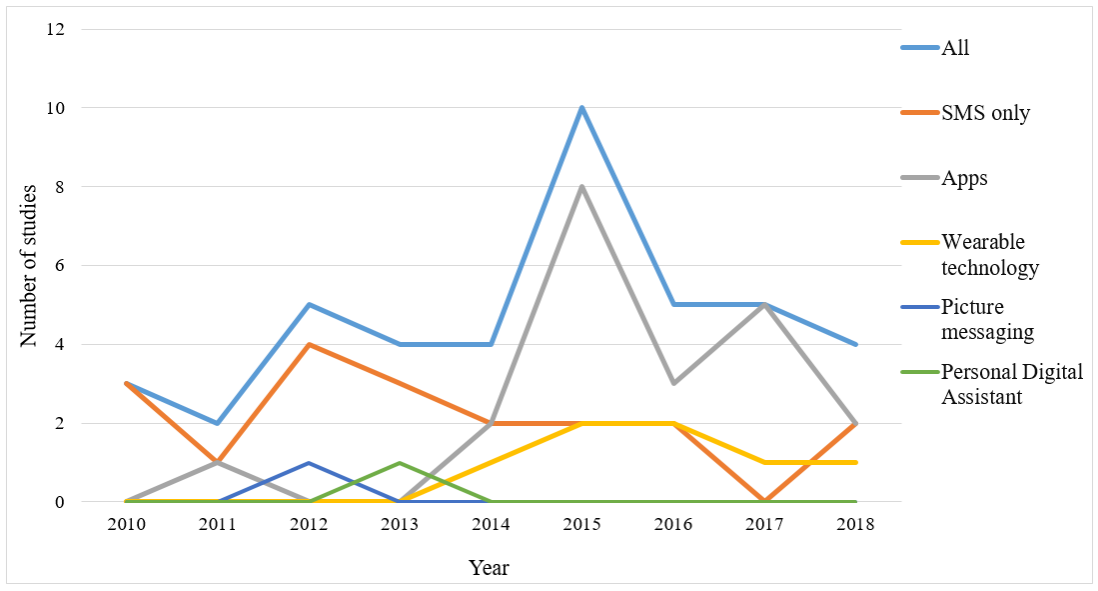
Number of eligible studies by year and mode of delivery.

The two predominant modes of intervention delivery via mHealth were SMS text messaging and mobile apps. The earliest studies identified were published in 2010 [41,42,62], and SMS remained the most studied form of mHealth for treatment in this population until 2014, at which time, apps subsequently overtook SMS in frequency reported in the published literature. Figure 2 outlines the overall number of studies identified by year and mode of delivery. SMS remained a popular component of evaluated mHealth interventions after 2015 but began to feature as secondary to other forms of mHealth tools including apps and wearable technology [51,61,68]. A total of 6 studies featured wearable technology as the primary mHealth component. Wearable technology goes hand in hand with mobile apps, which are often used for monitoring and collecting the data. Each of these 6 studies also featured at least one app. In all, 3 studies investigated the modes of mHealth other than SMS, apps, and wearables. In 2012, Woolford et al [34] explored an intervention based on *Photovoice*, where participants used picture messaging as part of the intervention, while Oliver et al [45] explored a novel method of self-monitoring using a personal digital assistant.

### Outcomes Measured

#### Adiposity-Related and Cardiometabolic Outcomes

For the studies assessing the effectiveness of interventions, the most commonly measured outcome was change in BMI z-score (alternatively referred to as standard deviation score) or BMI percentile using anthropometric measures, which 43% (18/42) of the included studies assessed (Multimedia Appendix 4). Herget et al [67] measured skinfold thickness too, whereas 4 studies reported additional clinical outcomes including blood pressure, biochemical samples, physical fitness, and insulin resistance [63-65,69,72].

#### Dietary Measures

In all, 31% (13/42) studies reported outcomes related to dietary intake or eating behavior; however, the measures used to assess these varied substantially, with nine specified and two unspecified outcome measures. The measures used included the Dutch Eating Behavior Questionnaire [44,62,79], 24-hour dietary food records [54], 3-day food records [58], photo-assisted 3-day food records, in addition to the Healthy Eating Index 2010 [46,80], food diaries [61], food frequency questionnaires [63,64,69], and items from the California Health Interview Survey [51,68,81]. Durrer et al [72] (a conference abstract) reported using sequential photogrammetry to measure eating speed. The study by Durrer et al [72] and another conference abstract by the same research team [53] also reported using *validated questionnaires* to assess eating disorders but failed to specify the exact measures. Finally, Pretlow et al [48] assessed (a) *whether participants could identify “problem foods” and withdraw from them*, (b) *whether participants were able to eliminate snacking*; and (c) *the extent to which participants were able to reduce the amounts of foods consumed at home meals* as part of their implementation of an addiction model, which was tracked using the mobile app.

#### Physical Activity

Overall, 4 of the studies included in this review assessed physical activity, objectively measured using accelerometers or wearable technology, as an outcome [46,54,58,72]. One study assessed physical activity by measuring engagement with a fitness app [66] and another by attendance at a high-intensity interval training program [67] (complemented by a variety of self-reported questionnaires). Additional self-reported measures included items on activity habits from the Youth Risk Behavior Surveillance Study [69] and physical activity and sedentary behavior items from the California Health Interview Survey [51,68].

#### Psychological Outcomes

A total of 6 studies that measured psychological health used a health-related quality of life (HRQoL) scale. The measures of HRQoL included the Child Health Questionnaire—PF50 [56], the Pediatric Quality of Life InventoryTM [60,65], the Perceived Quality of Life Scale Adolescents [51,68] and the KIDSCREEN 27 [67]. Further outcome measures related to psychological health and well-being included the Rosenberg Self-Esteem Scale [60], the Mental Health Inventory, the MacArthur Scale of Subjective Social Status, Harter Self Perception Profile for Adolescents, and sex-specific body dissatisfaction scales [63,64]. Chen et al [51,68] assessed self-efficacy using items from the Health Behavior Questionnaire, and Armstrong et al [69] (parent self-efficacy) using the Global Self-Efficacy scale, whereas Herget et al [67] measured self-efficacy, internalization of stigmatization, and perceived social support using validated questionnaires specified in the article. In addition to HRQoL, de Niet [56] measured self-perception using the Dutch version of the Self-Perception Profile for Children, which measures self-perceived competence. Kowatsch et al [70] measured the emotional and social relationship between the participants and their *chatbots*, using a short version of the attachment bond scale of the Working Alliance Inventory. The conference abstracts by Durrer et al [72] and Lallemand et al [53] reported the assessment of mental health, mood, and well-being as well as motivation, but the measure or measures used were not stated. Finally, Pretlow et al [48] also measured addiction guilt, stress, control, and self-esteem using individual self-report items.

#### Process Outcomes

We identified 3 studies which formally assessed the usability of the mHealth intervention. Oliver et al [45] used an adapted version of the System Usability Scale by Brooke [82] to assess their electronic dietary record, while Ptomey et al [46] administered Likert-scale questions on participant comfort using the tablet and its various features relevant to their specific intervention. O’Malley et al [39] measured technical effectiveness, technical efficiency, and usability via the Software Usability Measurement Inventory (SUMI) [83], which measures efficiency, effect, helpfulness, controllability, and learnability.

Acceptability was also widely measured, with 15 studies reporting patient experience of, or satisfaction with, the intervention. For the most part, this was assessed quantitatively using surveys, predominantly Likert-scales or similar rating-based survey items specifically designed for the individual studies [38,40,43,48,51,61,66,67]; however, Jensen et al [50] and Nguyen et al [73] used previously validated tools, the Client Satisfaction Questionnaire [84] and an adapted version of a satisfaction questionnaire by Golley et al [85]. In all, 8 studies assessed the acceptability of the intervention using qualitative methods [33-37,42,44,78], whereas an additional 5 studies included open-ended questions or interviews to supplement quantitative assessment of acceptability by collecting additional feedback [38,40,43,50,66].

Although adherence was the primary outcome for just 1 study [57], a further 12 studies reported adherence with the intervention as an outcome. Adherence was predominantly measured using the data for direct engagement with the technology [46,47,66,70], as well as responses to SMS communication [41,55,57,59,62], attendance at the face-to-face sessions [57,69], and the level of self-monitoring [25,50]. Nguyen et al [73] reported facilitator adherence to the program protocols (ie, fidelity) as well as participant engagement with the intervention as a whole; however, these were not specific to the mHealth component (SMS).

## Discussion

### Principal Findings

This review presents an overview of the literature on mHealth for pediatric weight management and its characteristics. Our study highlights substantial heterogeneity in the interventions, designs, participants, and outcomes assessed in studies that have evaluated the use of mobile technology for the treatment of childhood overweight and obesity. Our findings show that the majority of the work thus far has been aimed at assessing feasibility, acceptability, or usability of mHealth interventions for lifestyle treatment, with these forming parts of an emerging evidence base.

There is no doubt that current technology is rapidly developing, and this poses a difficult challenge for researchers to design and test technology using robust experimental methods while remaining relevant. This review reports that until recently, interventions based solely on text messaging comprised the extent of mobile therapeutic care in this population; however, there has been a sharp transition to apps, often complemented by wearable technology in the last 4 to 5 years. SMS remains a part of many interventions but is no longer necessarily considered the focus of research as is often the case with technological advances which have become embedded in practice [86]. Although our review shows that much of the recent work carried out was largely aimed at perfecting the technology and ensuring its feasibility in this population, it is clear that we can expect a continued rise in emerging evidence for its effectiveness in the near future as these studies give way to full trials. However, there is a possibility of marketing of mHealth interventions by commercial entities as *treatments* before completing the testing through experimental methods with the target end users. We identified only 1 systematic review [87] published in 2015, with only two included studies, that specifically met our inclusion criteria, indicating that this was until recently, a field in its infancy.

We investigated the literature specifically aimed at the treatment of childhood overweight and obesity using mHealth, which eliminated a large number of studies that also focused on prevention in this age group or included a high proportion of participants with normal weight. Many of the published systematic reviews have either included mHealth interventions for both prevention and treatment [88-90], or interventions aimed at specific behaviors associated with obesity (eg, sedentary behavior) [91,92]. However, owing to the complexity of overweight and obesity and the specific needs and challenges associated with treating rather than preventing excessive weight gain, we chose not to combine the two. Children with clinical obesity are a specific population where the accumulation of excess adipose tissue may already be affecting the body’s structures and functions and the child’s health and well-being. As such, obesity was defined as a disease by the WHO in the 1970s—a concept revisited more recently by the Childhood Task Force of the European Association for the Study of Obesity [93]. Given that obesity treatment involves a child or adolescent presenting to the health care system for support or help with this condition and related comorbidities, the type of interventions that may be effective are likely very different to that offered to a child for the prevention of obesity. In addition, clinical health services are more commonly required to address the treatment of obesity rather than its prevention and as such, we excluded prevention studies.

There is evidence that while an array of apps aimed at addressing childhood obesity exist, many lack inputs from health professionals with experience in treating childhood obesity or patients living with the condition [94]. A review of nutrition apps relevant to childhood weight management demonstrated that the majority of those available were not evidence based [95]. Our findings suggest that in the academic literature, BCT features prominently in many of the mHealth interventions developed, although in some cases these are components of BCTs such as self-monitoring, which when used in isolation may not be effective. We did not formally critically appraise the studies included in this review given our study design, but future reviews of interventions in this field should aim to do so, in particular, to evaluate the use and appropriateness of BCTs and their application to mHealth for this purpose. In addition, while outcomes including *usability* and *acceptability* were frequently measured within the studies we identified, there are no universally accepted definitions for these and the measures used for them were variable, often not validated at all or not validated for children. The authors are aware of ongoing Delphi consultations to develop a common definition and approach to measuring user experiences with pediatric electronic health interventions, which will be extremely useful in designing future interventions for this population.

The heterogeneous nature of mHealth is important to consider for comparing studies in the future. Automated text messaging, for example, is quite dissimilar to interactive apps. Furthermore, some types of mHealth are more closely related to other electronic means of treatment or telemedicine, and it may be more useful to compare the type of communication (eg, educational messages) rather than define these by the mode of communication (mobile device, email, or paper). Thus, limiting reviews to mobile devices means that intervention methods, which are similar but do not necessarily fall into the *mHealth* category (eg, Skype vs FaceTime counselling, or serious games for smartphones vs games consoles) may be counterproductive. Future reviews should possibly take into account this diverse nature of mHealth and aim to narrow meta-analyses, in particular to interventions which are more closely aligned.

The most common health-related outcome measure across all studies included in this review was BMI z-score or similar. Although change in BMI z-score provides a useful standardized proxy for change in adiposity, it is ultimately flawed as it represents size and shape rather than fat accumulation [96]. This remains a challenge that is universal to the study of childhood obesity. However, for future trials and reviews that assess the effectiveness of mHealth interventions, it is vital that attention is given to the definition of effectiveness, particularly where participants have multimorbidities and small changes in behavior or level of adipose tissue, which while not necessarily resulting in significant change in BMI, may still represent a positive outcome. For children and young people with unique needs and circumstances, such as those with autism spectrum disorder who are at increased risk of overweight and having secondary health issues [97], assessing BMI or indeed using general dietary measures such as food frequency questionnaires may not be useful. The studies in our review that assessed diet and physical activity did so using a very diverse set of measures, particularly for dietary intake. Diet is especially difficult to measure as doing so objectively is not practical and often not feasible and comparing tools with varying levels of accuracy and precision will present a challenge in assessing this as an outcome across studies. None of the measures of diet highlighted in this review are considered to be the most reliable [98]. Moreover, self-reported dietary intake measures have been demonstrated to be less reliable in children with higher BMI compared with those with lower BMI [99] and were developed for use in healthy adult populations. This will be an important consideration for critical appraisal of the evidence in future reviews, in addition to assessing adherence to the Consolidated Standards of Reporting Trials for eHealth (eCONSORT) [100] guidance for Web and mobile trials, which none of the studies included in this review referred to.

We did not identify any cost-effectiveness analyses of childhood obesity treatment via mHealth, which would provide important information to decision makers. Economic evaluations of such studies are needed because mobile technology is frequently cited as a *cost-effective* [37,101,102] alternative to in-person treatment in this population, in the absence of any actual study or formal assessment of the costs.

Finally, although none of the studies in our review observed adverse events related to the use of the mobile device, future studies will need to report on unintended events in a systematic manner and be mindful of whether mHealth interventions increase the risk of negative effects such as exposure to digital marketing or safety concerns because of distraction (eg, road traffic accidents while using devices) [103].

### Strengths and Limitations

This study has a number of strengths. Our focus on participants with overweight and obesity specifically, while including a broad range of study designs, has allowed us to provide a comprehensive overview of the literature at all stages of the research process in relation to mHealth for the treatment of childhood overweight or obesity. This is the first review to do so to date. This will provide a useful basis for researchers and health care professionals to identify gaps in the literature and areas for development or to facilitate defining clear questions regarding the effectiveness of interventions on specific outcomes of interest (eg, measures of usability or body composition). There are also some limitations. We did not include Web 2.0 interventions, such as those utilizing Facebook, which participants would often use on a mobile device. As we cannot guarantee the usage on a mobile device, it was not possible to distinguish interventions that were exclusively used on a mobile device from those that might be used on desktop computers. We aimed to minimize the publication bias by including gray literature and conference proceedings; however, it is possible that some conference abstracts were not identified using our search methods given the varying levels of indexing for these. Some bias may also have arisen from our exclusion of studies not published in English. In addition, while a second reviewer thoroughly screened a portion of all titles and abstracts and full texts, the entire set of search results was only screened by one reviewer. The initial large percentage of disagreement in studies to include may, however, represent bias on the part of the reviewers or indeed ambiguity in the inclusion criteria, although the team addressed this using the best available means, through discussion.

### Conclusions

In summary, the field of mHealth for the treatment of childhood overweight and obesity is new, and the evidence base is still emerging; however, it is certainly a rapidly developing research area. The studies to date have mainly aimed at assessing the feasibility of interventions and are heterogeneous in nature with a diverse variety of outcome measures used. There is a need for cost-effectiveness studies alongside further large, robust RCTs, which employ valid outcome measures and report following eCONSORT guidelines.

## Appendix

Multimedia Appendix 1 PubMed search strategy.

Multimedia Appendix 2 Full inclusion and exclusion criteria.

Multimedia Appendix 3 Decision tool for screening.

Multimedia Appendix 4 Table of included studies.

## Abbreviations

BCT: behavior change theory
eCONSORT: Consolidated Standards of Reporting Trials for eHealth
HRB: Health Research Board
HRQoL: health-related quality of life
ICT: information and communication technology
IDD: intellectual or developmental disabilities
mHealth: mobile health
RCT: randomized controlled trial
WHO: World Health Organization
