# Supplementary material for: Mobile Health for Pediatric Weight Management: Systematic Scoping Review
Source: JMIR Mhealth Uhealth. 2020 Jun 3;8(6):e16214. doi: 10.2196/16214 (PMC7301268; doi:10.2196/16214)
Supplement: Multimedia Appendix 1 [file mhealth_v8i6e16214_app1.docx]

# PubMed search strategy

| **PubMed** | | |
| --- | --- | --- |
| #4 | #1 AND #2 AND #3 Filters: **Publication date from 2000/01/01 to 2018/12/31; Humans** | 1,752 |
| #3 | Search ((((((microcomputer OR telemedicine[MeSH Terms])) OR (microcomputer[Title/Abstract] OR telemedicine[Title/Abstract] OR personal digital assistant[Title/Abstract] OR digital health[Title/Abstract] OR wireless[Title/Abstract] OR smartphone[Title/Abstract] OR smart phone[Title/Abstract] OR cell phone[Title/Abstract] OR cellphone[Title/Abstract] OR mobile phone[Title/Abstract] OR handheld[Title/Abstract] OR mhealth[Title/Abstract] OR m-health[Title/Abstract] OR app[Title/Abstract] OR tablet computer[Title/Abstract] OR tablet PC[Title/Abstract] OR iPad[Title/Abstract] OR messaging[Title/Abstract] OR messages[Title/Abstract] OR eHealth[Title/Abstract] OR e-health[Title/Abstract] OR electronic health[Title/Abstract] OR telehealth[Title/Abstract] OR connected health[Title/Abstract] OR internet[Title/Abstract])) OR (mobile[Title/Abstract] AND app[Title/Abstract])) OR (mobile[Title/Abstract] AND electronic[Title/Abstract] AND device[Title/Abstract])) OR (mobile[Title/Abstract] AND health[Title/Abstract])) OR (mobile[Title/Abstract] AND application[Title/Abstract]) Sort by: Author | 181,111 |
| #2 | Search ((child OR adolescent OR minors[MeSH Terms])) OR (Child*[Title/Abstract] OR Adolescen*[Title/Abstract] OR Teen*[Title/Abstract] OR Youth[Title/Abstract] OR Minors[Title/Abstract] OR Pediatric[Title/Abstract] OR Paediatric[Title/Abstract] OR Young[Title/Abstract]) Sort by: Author | 3,661,119 |
| #1 | Search (obesity[MeSH Terms]) OR (obesity OR obese OR overweight OR weight loss OR BMI OR body mass index OR body weight OR weight management[MeSH Terms]) Sort by: Author | 892,322 |
