# Supplementary material for: Mobile Health for Pediatric Weight Management: Systematic Scoping Review
Source: JMIR Mhealth Uhealth. 2020 Jun 3;8(6):e16214. doi: 10.2196/16214 (PMC7301268; doi:10.2196/16214)
Supplement: Multimedia Appendix 2 [file mhealth_v8i6e16214_app2.docx]

# Inclusion & exclusion criteria

| **Inclusion** | **Exclusion** |
| --- | --- |
| - Studies assessing effectiveness of lifestyle interventions which use mobile electronic devices for weight management in children - Studies whereby mobile electronic devices are the primary mode of delivery for at least one study phase *or* where the mobile component is independently assessed - Interventions aimed at reducing adiposity or related clinical measures in children with overweight/obesity, or maintaining weight following treatment for overweight/obesity - Qualitative, quantitative or mixed-methods studies exploring participant experience in partaking in such an intervention - Studies assessing fidelity/process evaluations or economic evaluations of such interventions - Studies whereby participants include predominantly children aged ≤17 years with overweight or obesity | - Non-mHealth forms of telemedicine such as use of computer-, email-, or telephone-only interventions - Studies aimed at preventing obesity in children of normal weight - Studies whereby participants are in-patients - Interventions aimed at management of underweight - Articles describing interventions or mobile platforms which do not discuss any kind of evaluation involving patients - Studies whereby mHealth is a minor part of a broader intervention and is not independently assessed (eg SMS reminders for a predominantly face-to-face intervention) - Studies where the only mHealth component is a wearable device which is used for measuring outcomes only (eg accelerometers) - Studies assessing treatment related to pharmacotherapy or bariatric surgery - Studies published prior to the year 2000 |
