# Supplementary figures and images for: Mobile Health for Pediatric Weight Management: Systematic Scoping Review
Source: JMIR Mhealth Uhealth. 2020 Jun 3;8(6):e16214. doi: 10.2196/16214 (PMC7301268; doi:10.2196/16214)

# Decision tool for screening


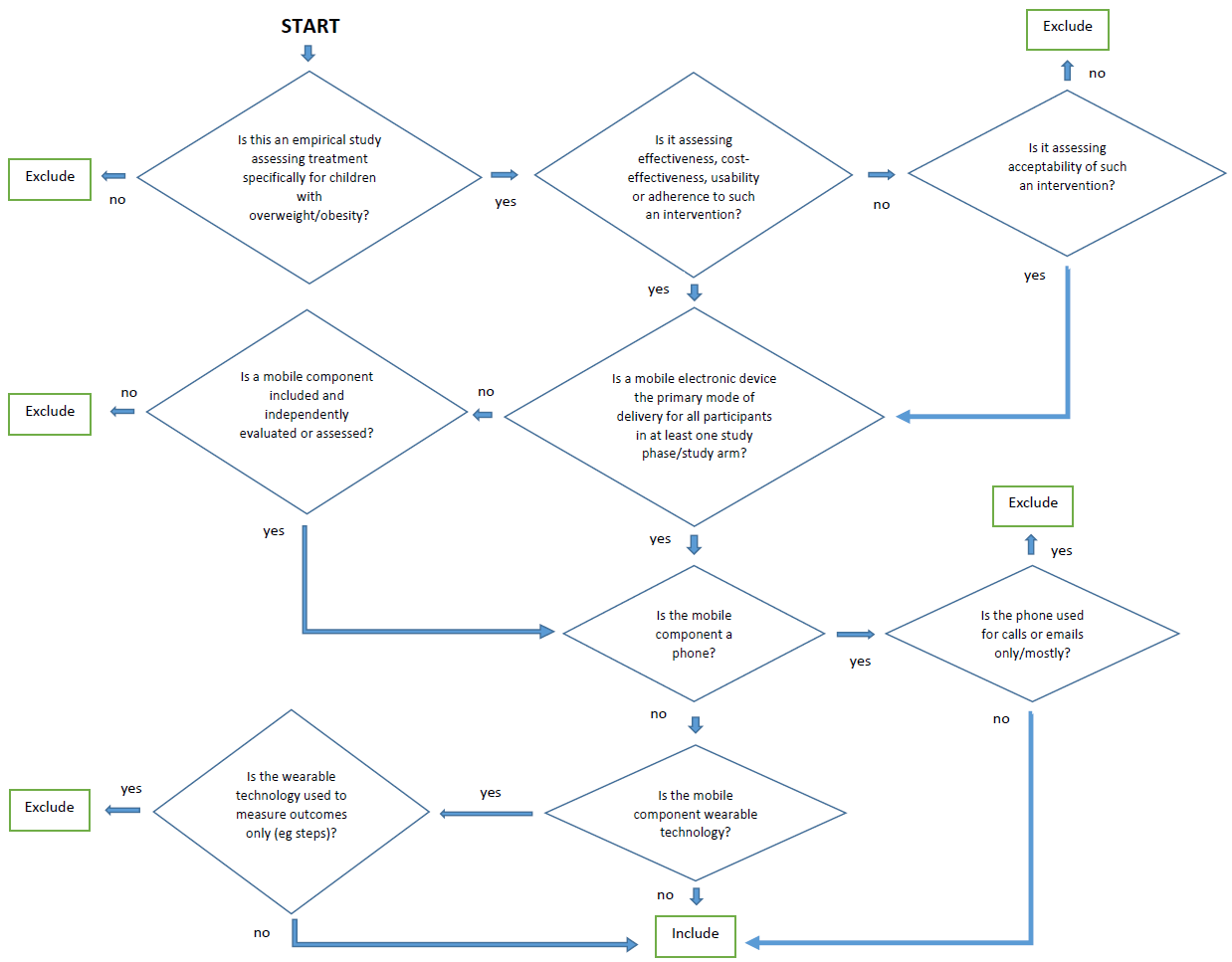

Supplement: Multimedia Appendix 3 [file mhealth_v8i6e16214_app3.docx]
