# Supplementary material for: Mobile Health for Pediatric Weight Management: Systematic Scoping Review
Source: JMIR Mhealth Uhealth. 2020 Jun 3;8(6):e16214. doi: 10.2196/16214 (PMC7301268; doi:10.2196/16214)
Supplement: Multimedia Appendix 4 [file mhealth_v8i6e16214_app4.docx]

**Table 2.** Included studies.

| Study | Design | Participants | Nature of mobile health | Outcomes measured | Behavior change theory |
| --- | --- | --- | --- | --- | --- |
| Bauer et al, 2010 [42] | Feasibility study | 7-12-year-olds with overweight or obesity (n=40) | SMS | BMI-SDS^a^ and adherence^b^ | Self-monitoring [75] |
| Kornman et al, 2010 [63] | RCT^c^ | 13-16-year-olds with overweight or obesity (n=49) | SMS | Engagement with SMS (responses) | Social Cognitive Theory |
| Woolford et al, 2010 [43] | Feasibility study | 12-18-year-olds with overweight or obesity (≥95th percentile) (n=20) | SMS | Technology performance and acceptability^d^ | Message framing for motivation [76], varying messages [77] |
| Cushing et al, 2011 [35] | Mixed method | 14-18-year-old females with overweight or obesity (≥85th percentile) (n=3) | Personal electronic device (iPod touch) | Adherence and usability^e^ | Self-monitoring |
| Woolford et al, 2011 [33] | Qualitative | 11-19-year-olds with overweight or obesity (≥98th percentile) (n=24) | SMS | Acceptability of SMS message content | Social Determination Theory and Elaboration Likelihood Model for motivational interviewing |
| de Niet et al, 2012 [58] | RCT | 7-12-year-olds with overweight or obesity (n=141) | SMS | Dropout, adherence to SMS, and BMI-SDS | Self-monitoring |
| de Niet et al, 2012 [57] | RCT | 7-12-year-olds with overweight or obesity (n=141) | SMS | BMI-SDS, eating behavior, and psychological well-being (competence, self-esteem, and quality of life) | Self-monitoring |
| Nguyen et al, 2012 [65] | RCT | 13-16-year-olds with overweight or obesity (BMI z-score: 1.0–2.5) (n=151) | SMS | BMI z-score, eating behavior (FFQ^f^), psychosocial well-being, and anthropometric and metabolic measures | Social Cognitive Theory |
| Woolford et al, 2012 [34] | Qualitative | 13-19-year-olds with overweight or obesity (≥96th percentile) (n=23) | Picture messaging (Photovoice) | Acceptability | Not specified |
| Fortune et al, 2012 [56] | Pilot study | 12‐18-year-olds with BMI>85th centile (n=165) | SMS | Adherence (responses and engagement) and feasibility | Goal setting |
| Nguyen et al, 2013 [64] | RCT | 13-16-year-olds with overweight or obesity (BMI z-score: 1.0–2.5) (n=151) | SMS | BMI z-score, eating behavior, psychosocial well-being, and anthropometric and metabolic measures | Social Cognitive Theory |
| Oliver et al, 2013 [46] | Feasibility study | 9-15-year-olds with overweight or obesity, seeking obesity treatment (n=30) | Personal digital assistant | Acceptability and usability | Self-monitoring |
| Patrick et al, 2013 [61] | RCT | 12-16-year-olds with overweight or obesity (≥85th percentile) + two risk factors for T2DM^g^ (n=101) | SMS | BMI z-score, health-related quality of life, and self-esteem | Transtheoretical model |
| Sharifi et al, 2013 [37] | Qualitative | Parents (n=38) of children aged 6-12 years with overweight or obesity | SMS | Acceptability and preferences | None specified |
| Kim et al, 2014 [38] | Mixed method | 13-29-year-olds (n=6 user testing, n=24 for pilot) with BMI ≥85th percentile | App (iPod touch) | Acceptability and usability | Motivational interviewing |
| O'Malley et al, 2014 [40] | Usability study | 12-17-year-olds with overweight or obesity (≥98th centile) (n=10) | App | Usability (technical efficiency, effect, helpfulness, controllability, and learnability) | Social Cognitive Theory, the Theory of Planned Behavior, and the Capability, Opportunity, Motivation, Behavior (COM-B)Model |
| Smith et al, 2014 [36] | Qualitative | 12-16-year-olds with overweight or obesity (n=12) and their parents (n=12) | SMS | Acceptability | Self-determination theory and goal-setting theory |
| Straker et al, 2014 [59] | Waitlist controlled trial | 12-16-year-olds with overweight or obesity (n=69) and their parents | SMS | Physical activity, diet, and BMI z-scores | Self-determination theory and goal setting |
| Xu et al, 2014 [48] | Pilot study | Children aged 11-14 years (n=6) with overweight or obesity (BMI ≥90th percentile) | App | Adherence and acceptability | Feedback loops |
| Buchter et al, 2015 [53] | Pilot study | Children (n=6) with severe obesity (BMI≥99.5, median BMI z-score 3.0, age 13.2 years, SD 2.3 years) | Mobile health information system for tablet | BMI-SDS | Not specified |
| Davis et al, 2015 [55] | Pilot study | Families (n=12) participating in a family-based behavioral group program for obesity | App (tablet) | Physical activity, diet, and BMI z-score (children) or BMI (parents) | Not specified |
| Durrer et al, 2015 [73] | Longitudinal field study | 13–17-year-olds with overweight or obesity (≥97th percentile) (n=6) | Apps (tablet) and wearable technology (FitBit) | Well-being, mental health, mood, eating disorders, body weight and BMI-SDS, blood pressure, speed, of eating, physical activity, and degree of relaxation | Not specified |
| Lallemand et al, 2015 [54] | Pilot study | 13-17-year-olds with severe obesity (n=6) | App | Eating disorders, physical and mental health, well-being, motivation, and parenting | Not specified |
| Nguyen et al, 2015 [74] | RCT process evaluation | 13-16-year-olds with overweight or obesity (BMI z-score: 1.0–2.5) (n=151) | SMS | Facilitator adherence and delivery dose, participant involvement and interaction, and acceptability | Social Cognitive Theory |
| O'Malley et al, 2015 [66] | RCT | 12-17-year-olds with overweight or obesity (≥98th centile) (n=134) | App | BMI-SDS, anthropometric and clinical biomarkers, and health-related quality of life | Social Cognitive Theory, the Theory of Planned Behavior, and the Capability, Opportunity, Motivation, Behavior (COM-B)Model |
| Pretlow et al, 2015 [49] | Pilot study | 10-21-year-olds with overweight or obesity (≥85th percentile) (n=43) | App and SMS | BMI, self-esteem, control over food, stress eating, addiction guilt, stress, control, self-esteem, and acceptability | Addiction treatment approach |
| Price et al 2015 [60] | RCT | Parents (n=160) of children aged 6-12 years with a BMI ≥95th percentile | SMS | Engagement with SMS | Social Cognitive Theory |
| Ptomey et al, 2015 [47] | Pilot study | 11-18-year-olds with overweight or obesity (≥85th percentile) and mild intellectual or developmental disabilities (n=20) | Apps (iPad) and FitBit | BMI, physical activity, diet, adherence, and acceptability | Not specified |
| Herget et al, 2016 [68] | RCT | 13-18-year-olds with overweight or obesity (BMI ≥90th percentile according to German reference values) (n=28) | SMS | Program attendance, BMI-SDS, anthropometric and metabolic measures, physical exercise or sedentary behavior, health-related quality of life, self-efficacy, internalization of stigmatization, perceived social support, and outcome expectations over physical activity, and acceptability | Social Cognitive Theory |
| Jensen et al, 2016 [51] | Pilot study | 13-17-year-olds with overweight or obesity (BMI percentile ≥85%) (n=16) and their parent or guardian | App and SMS | BMI z-scores and anthropometrics, adherence to self-monitoring, and acceptability | Not specified |
| Kulendran et al, 2016 [50] | Pilot study | 14-year-olds attending a weight-loss camp (n=27) | SMS | BMI (maintenance) | Commitment devices [86] |
| Mameli et al, 2016 [62] | RCT | 10-17-year-olds with overweight or obesity (BMI≥95th percentile) (n=43) | App, wearable technology, and SMS | BMI-SDS, diet, level of commitment to the intervention, acceptability, awareness on the importance of lifestyle changes, and change of habits using the intervention | Not specified |
| Ptomey et al, 2016 [39] | Qualitative | Parents (n=18) of 11-18-year-olds, with overweight or obesity and mild intellectual or developmental disabilities | Apps (iPad) and FitBit | Acceptability | Not specified |
| Chen et al, 2017 [52] | Pilot study | Chinese American 13-18-year-olds (n=40) with overweight or obesity (BMI≥85th percentile) | SMS, wearable technology, and apps | BMI percentile, diet, physical or sedentary activity, quality of life, physical activity self-efficacy and healthy eating self-efficacy, and acceptability | Social Cognitive Theory |
| Gabrielli et al, 2017 [45] | Feasibility study | 7-12-year-olds (n=6), classified as overweight (BMI 85th-94th percentile) | App | Usability and acceptability | Transtheoretical model |
| Kowatsch et al, 2017 [41] | Usability study | Children presenting for obesity treatment (n=11) | App | Usability and acceptability | Not specified |
| Kowatsch et al, 2017 [71] | RCT | Children presenting for obesity treatment (n=15) | App | Adherence, emotional and social relationship between patient and technology | Not specified |
| Tripicchio et al, 2017 [67] | Pre-post study | 2-18-year-olds with overweight or obesity (≥85th percentile) (n=64) | App (tablet) | BMI z-scores, attendance, engagement, acceptability, and open-ended feedback | Goal setting and personalized feedback |
| Armstrong et al, 2018 [70] | RCT | 5-12-year-olds (n=101) with overweight or obesity (BMI ≥95th percentile) and their parents | SMS | BMI z-scores, child health behaviors, cardiovascular fitness, parent BMI and self-efficacy for change, and adherence to clinic visits | Motivational interviewing |
| Chen et al, 2018 [69] | RCT | Chinese American 13-18-year-olds with overweight or obesity (BMI≥85^th^ percentile) (n=40) | SMS, wearable technology, and apps | BMI percentile, diet, physical activity, sedentary activity, quality of life, and physical activity self-efficacy and healthy eating self-efficacy | Social Cognitive Theory |
| Heldt et al 2018 [72] | RCT | 11-17-year-olds with overweight or obesity (BMI-SDS 2.56, SD 1.7-3.5) (n=22) | App | Engagement (use of the app) | Not specified |
| Saez et al, 2018 [44] | Feasibility study | 13-18-year-olds with overweight or obesity (n=262) | SMS | Reach and acceptability | The Reader-to-Leader Framework |

^a^BMI-SDS: body mass index standard deviation score.

^b^Level of adherence to the intended intervention components by participants.

^c^RCT: randomized controlled trial.

^d^Level to which the intervention is acceptable to the intended end user.

^e^Technical effectiveness, efficiency, and/or satisfaction with the intervention by the intended end user.

^f^FFQ: Food Frequency Questionnaire.

^g^T2DM: type II diabetes mellitus.
